# Supplementary material for: Uncleaved prefusion-optimized gp140 trimers derived from analysis of HIV-1 envelope metastability
Source: Nat Commun. 2016 Jun 28;7:12040. doi: 10.1038/ncomms12040 (PMC4931249; doi:10.1038/ncomms12040)
Supplement: Supplementary Information — Supplementary Figures 1-14 and Supplementary Table 1 [file ncomms12040-s1.pdf]

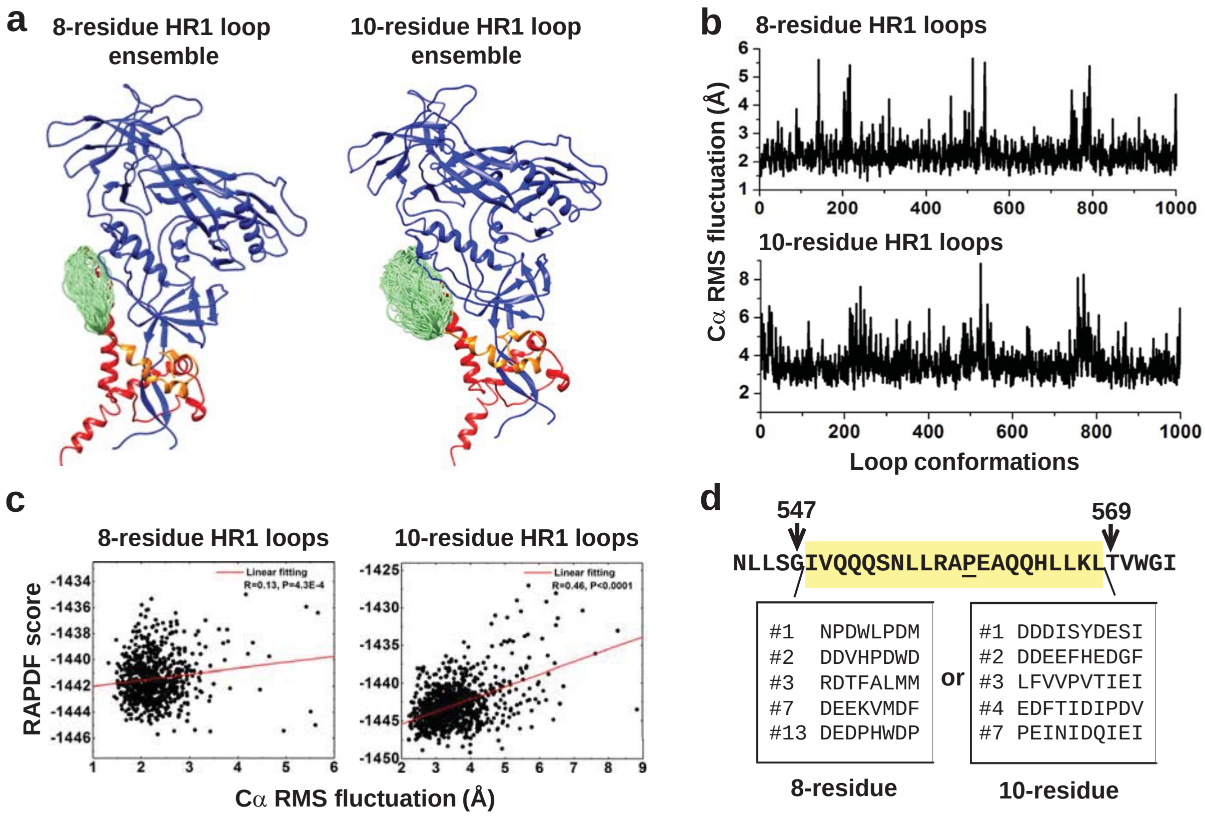

Supplementary Figure 1. Ensemble-based protein design of the HR1 region (547-569) with loop lengths of 8 and 10 residues. (**a**) Conformational ensembles of 8- (left) and 10-residue (right) HR1 loops colored in green with gp120 and two gp41$_{ECTO}$ regions (518-547 and 569-664) within one gp140 protomer colored in blue, orange, and red, respectively. (**b**) Cα root-mean-square (RMS) fluctuation of 8- (upper panel) and 10-residue (lower panel) redesigned HR1 loops. (**c**) Correlation between RAPDF score and Cα RMS fluctuation determined for 8- (left) and 10-residue (right) redesigned HR1 loops. (**d**) 5 top-ranking sequences manually selected for HR1 redesign between residues G547 and T569 using an 8- (left) or 10-residue (right) connecting loop. The region in WT BG505 SOSIP.664 that was subjected to computational design is highlighted in yellow.

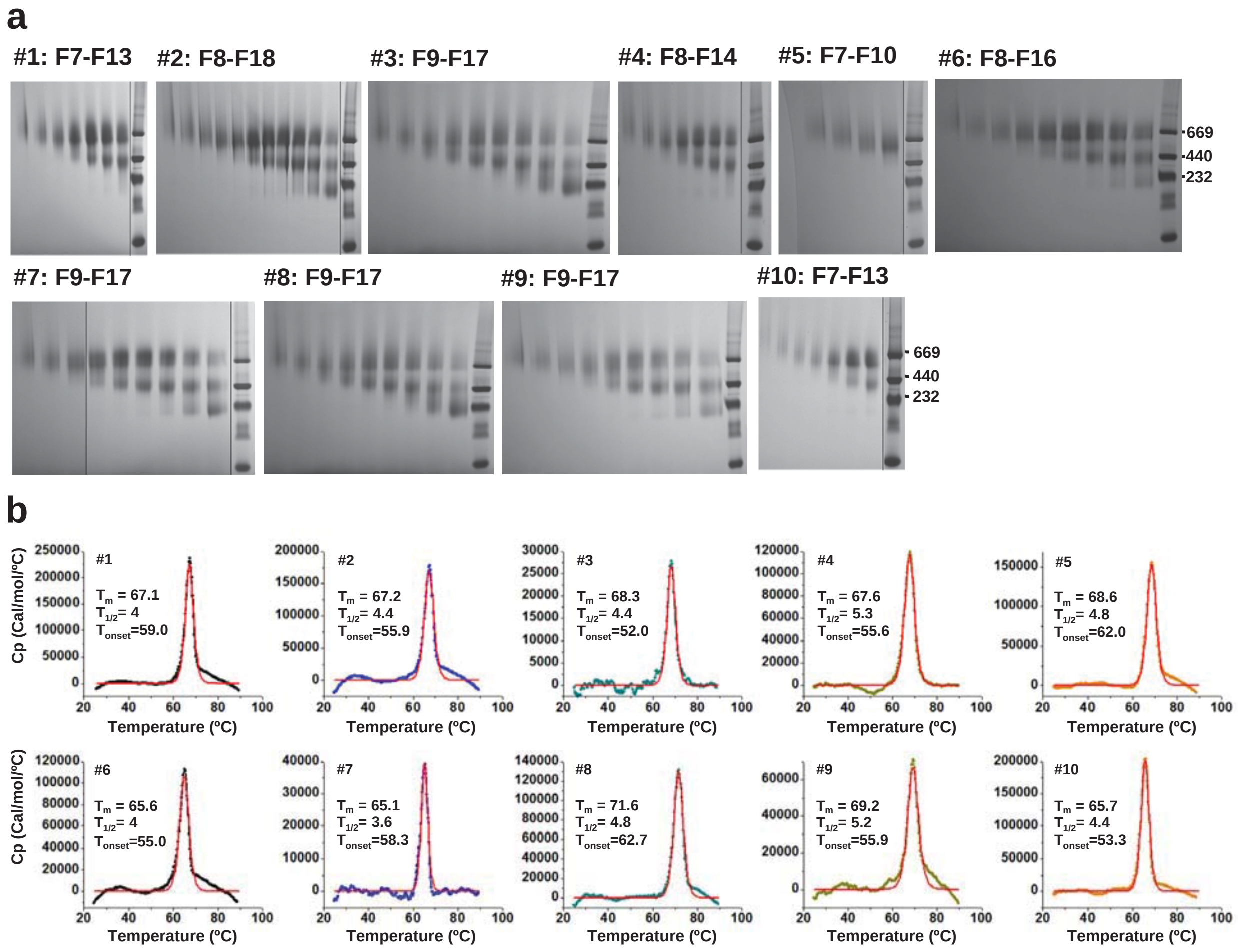

Supplementary Figure 2. Biochemical and biophysical characterization of 10 HR1 redesigns. (**a**) BN-PAGE of 293 F-expressed, GNL-purified proteins for 10 HR1-redesigned BG505 SOS.664 gp140 constructs after SEC on a Superdex 200 10/300 column. The SEC fractions tested are indicated above the gel images. (**b**) Thermal stability of 10 HR1-redesigned BG505 SOS.664 gp140 trimers measured by differential scanning calorimetry (DSC). Three thermal parameters (T_m, $T_{1/2}$ and T_{onset}) are labeled on the DSC profiles. Data fitting was performed using the standard fitting software provided by the vendor. Of note, the fluctuation of T_{onset} values may be partially attributed to the use of Superdex 200 10/300 column, which has less separation power than HiLoad Superdex 200 16/600 PG column in trimer purification.

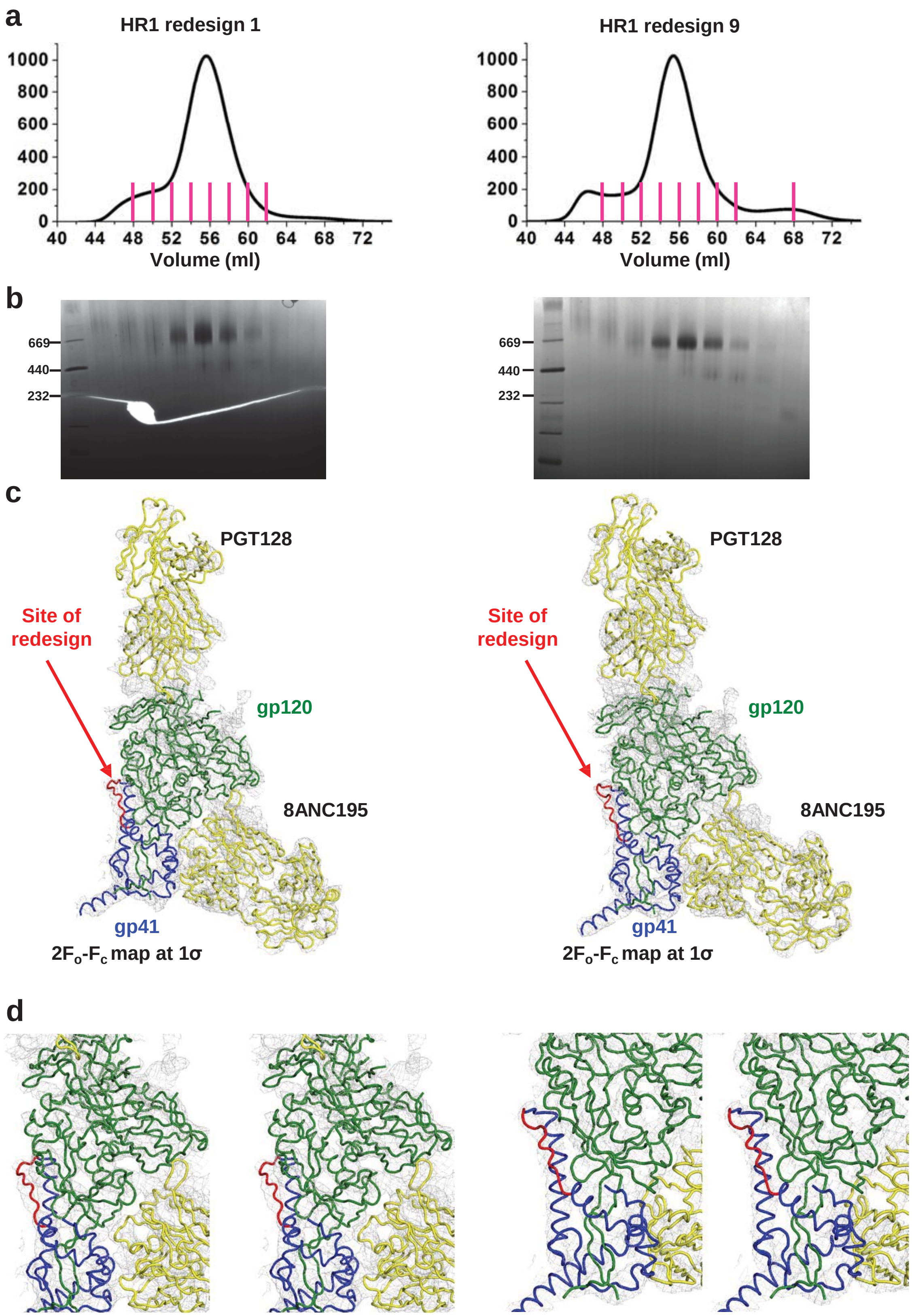
a
HR1 redesign 1
HR1 redesign 9
1000
800
600
400
200
0
40 44 48 52 56 60 64 68 72
Volume (ml)
b
669
440
232
c
PGT128
Site of redesign
gp120
8ANC195
gp41
2F$_o$-F$_c$ map at 1σ
d

Supplementary Figure 3. Crystallographic analysis of BG505 HR1 redesigns 1 and 9. (**a**) SEC profiles of 293 S-expressed, 2G12-purified BG505 HR1 redesigns after SEC on a HiLoad Superdex 200 16/600 PG column. The fractions selected for BN-PAGE, DSC analysis, and crystallization are labeled on the SEC traces with magenta lines. (**b**) BN-PAGE of SEC fractions for HR1 redesigns 1 (left) and 9 (right). (**c**) Crystal structures of HR1 redesigns 1 (left) and 9 (right) in complex with PGT128 and 8ANC195 Fabs (in yellow). The backbone traces of gp120, gp41, and the redesigned HR1 loop are colored in green, blue, and red, respectively. The 2Fo-Fc map is contoured at 1 σ as a grey mesh. (**d**) Stereo images of $2F_o$-F_c electron density maps (gray mesh) of HR1 redesigns 1 and 9 crystal structures. The color coding scheme is the same as in (c).

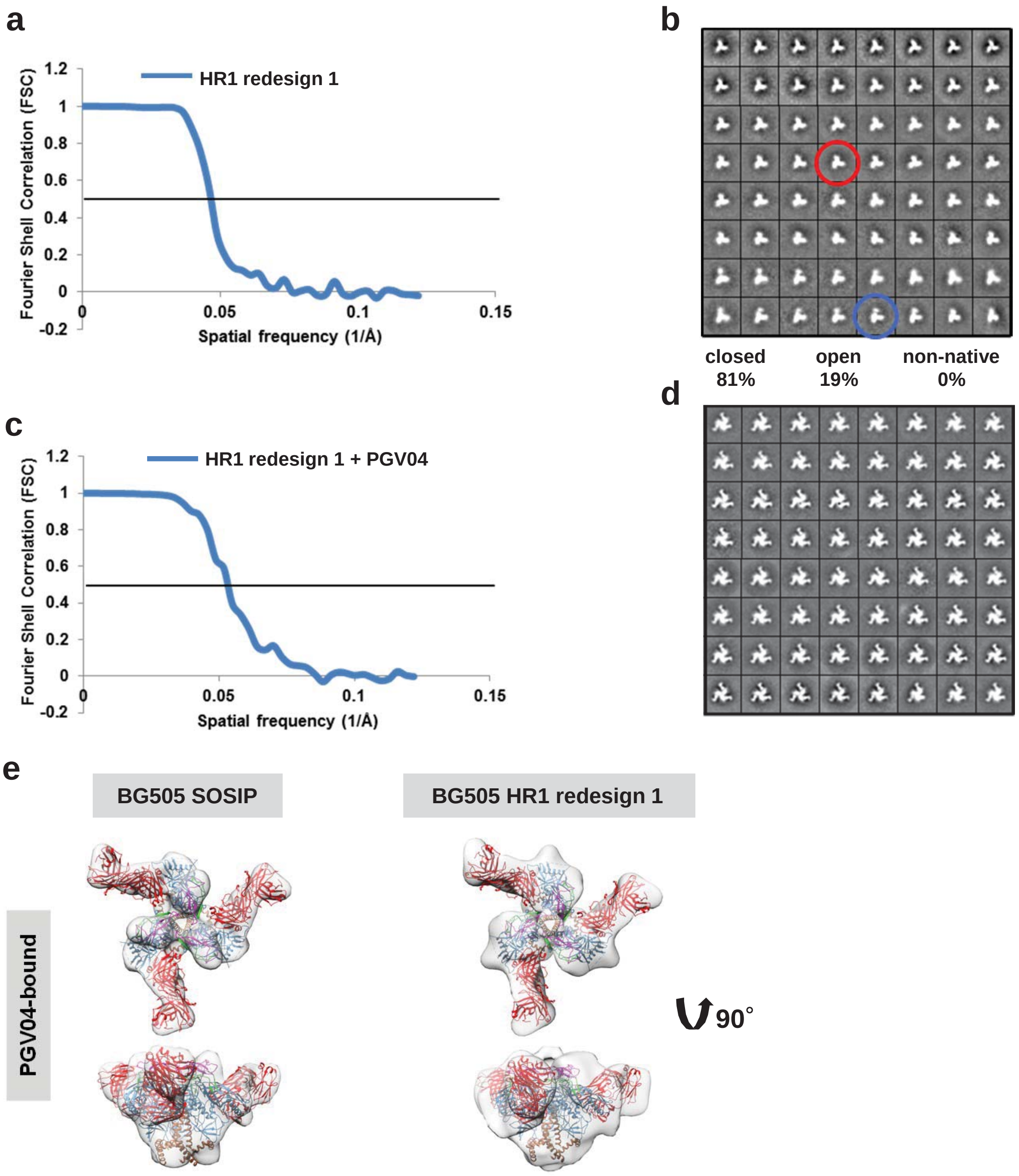

Supplementary Figure 4. Negative-stain EM of BG505 HR1 redesign 1. (**a**) The estimated resolution (22 Å) of the EM reconstruction for HR1 redesign 1 was calculated from the Fourier shell correlation (FSC) using a cut-off of 0.5. (**b**) Reference-free 2D class averages from negative-stain EM of HR1 redesign 1 trimer. To analyze the quality of the trimers, we calculated the percentages of closed native-like trimers (red circle), partially open native-like trimers (blue circle), and non-native trimers. (**c**) The final estimated resolution for HR1 redesign 1 trimer bound to Fab PGV04 was 19 Å. (**d**) Reference-free 2D class averages from negative-stain EM of HR1 redesign 1 trimer bound to Fab PGV04. (**e**) Top and side views of the 3D reconstruction EM densities of PGV04-bound BG505 SOSIP.664 trimer (left) and HR1 redesign 1 (right). The trimer densities are shown in gray with the high-resolution cryo-EM structure of BG505 SOSIP.664 trimer (PDB 3J5M, gp120 in blue with V1V2 in magenta, V3 in green, gp41 in brown) in complex with PGV04 (red) fitted into the density.

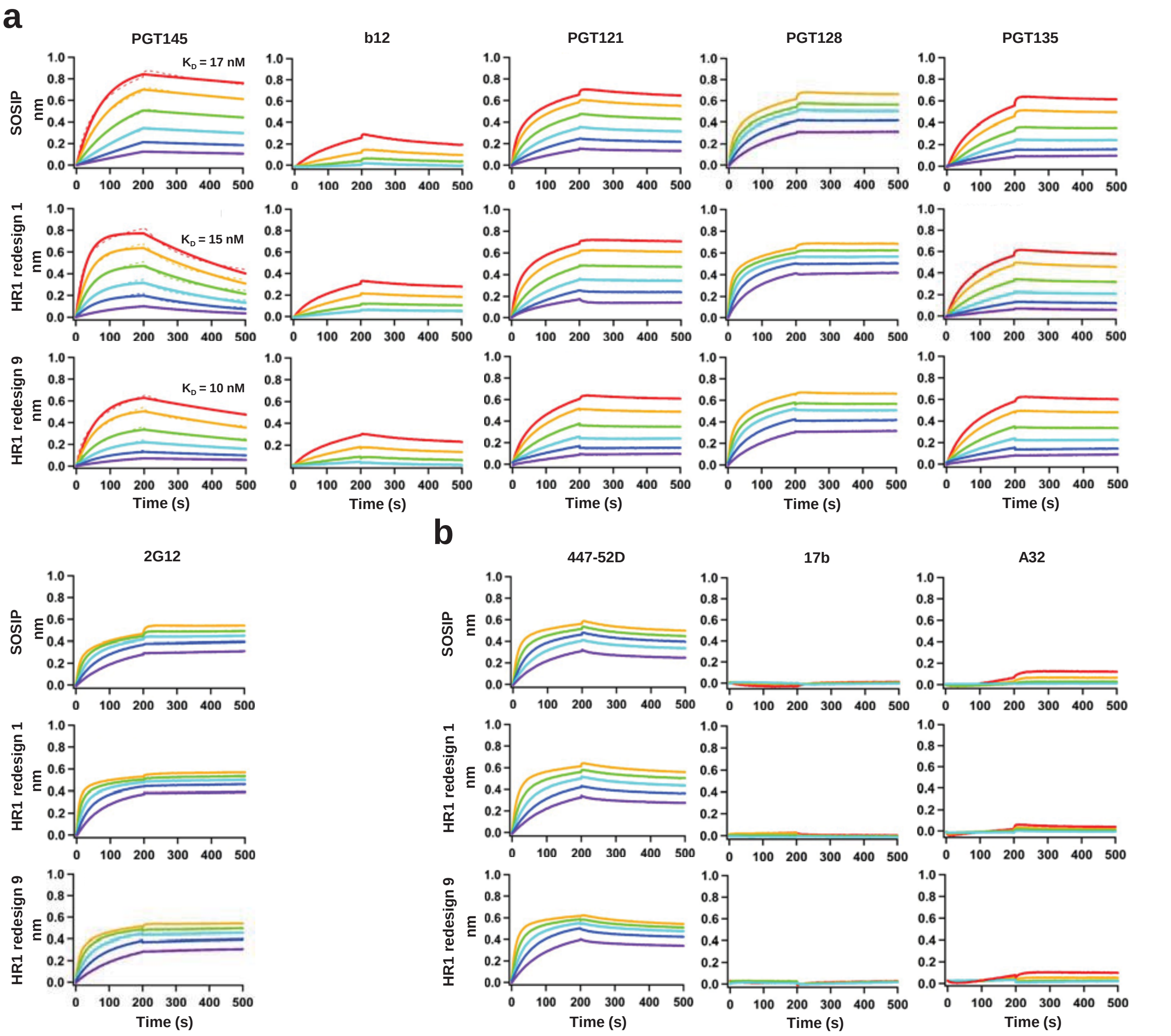

Supplementary Figure 5. Additional antigenic profiles for BG505 SOSIP.664 trimer and two HR1 redesigns. Profiles of Env trimer binding to bNAbs and non-NAbs are shown in (**a**) and (**b**), respectively. Sensorgrams were obtained from an Octet RED96 instrument using a titration series of six trimer concentrations (200-12.5 nM by 2-fold dilution). K_D values calculated from 1:1 global fitting are labeled for the V1V2 apex-directed bNAb PGT145 in (a).

a

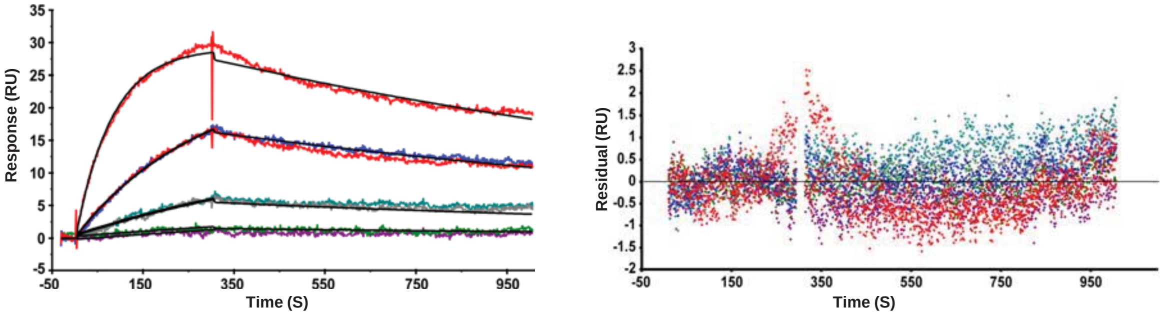

k_a	6.01±0.02e4 1/Ms	k_d	5.79±0.03e-4 1/s
Rmax	29.30±0.05 RU		
K_A	1.04e8 1/M	K_D	9.64 nM
Chi2	0.299		

b

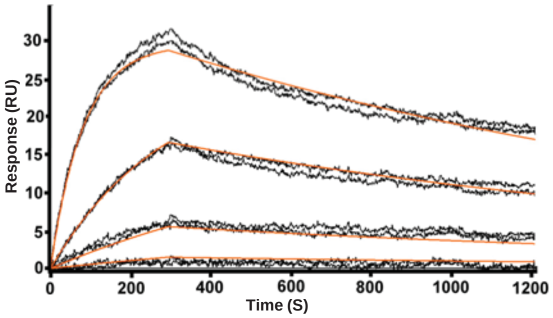

k_a	6.30e4 1/Ms	k_d	5.24e-4 1/s
K_D	8.34 nM		

Supplementary Figure 6. Kinetics of 2G12-purified BG505 SOSIP.664 trimer binding to V3-directed non-NAb 19b determined by surface plasmon resonance (SPR). Binding was performed on a BIACORE 3000 instrument (GE Healthcare) equipped with a research-grade CM3 sensor chip. The ligand, 19b IgG (150 kDa), was immobilized using NHS/EDC amine-coupling chemistry. The surface of two flow cells were activated for 7 min with a 1:1 mixture of 0.1 M NHS (N-hydroxysuccinimide) and 0.1 M EDC (3-(N,N-dimethylamino) propyl-N-ethylcarbodiimide) at a flow rate of 5 ul/min. The ligand resuspended in 10 mM sodium acetate (pH 5.5) was immobilized at a density of 50 RU on flow cell 2, whereas flow cell 1 was immobilized with a non-related antibody at the same density to serve as a reference surface. All surfaces were blocked with a 7 min injection of 1 M ethanolamine-HCl, pH 8.5. To collect kinetic data, the analyte, BG505 SOSIP.664 (520 kDa) in HBS-EP+ buffer (10mM HEPES, 150 mM NaCl, 0.05% P20, pH 7.4), was injected over the two flow cells at 200, 50, 12.5, and 3.125 nM at a flow rate of 20 ul/min (no mass transport limitation observed at this flow rate) at 25 °C. The complex was allowed to associate and dissociate for 300 and 900s, respectively. Duplicate injections (in random order) of each analyte sample and buffer blank were flowed over the two surfaces. The surface was regenerated by injection of 10 mM glycine-HCl (pH 1.5) for 30s between cycles. Data were collected at a high rate setting and were fit to a 1:1 Langmuir interaction model (red) using the global data analysis by (**a**) BIAevaluation software (ver. 4.1) and (**b**) Scrubber software (ver. 2.0). The accuracy of fitting was confirmed for the data derived from BIAevaluation (**a**, right) by plotting residual (discrepancy between experimental and calculated data points) against time, which showed residuals randomly scattered along the X-axis and within the same order of magnitude as the instrumental noise (≤ 2 RU), as expected for a good fit.

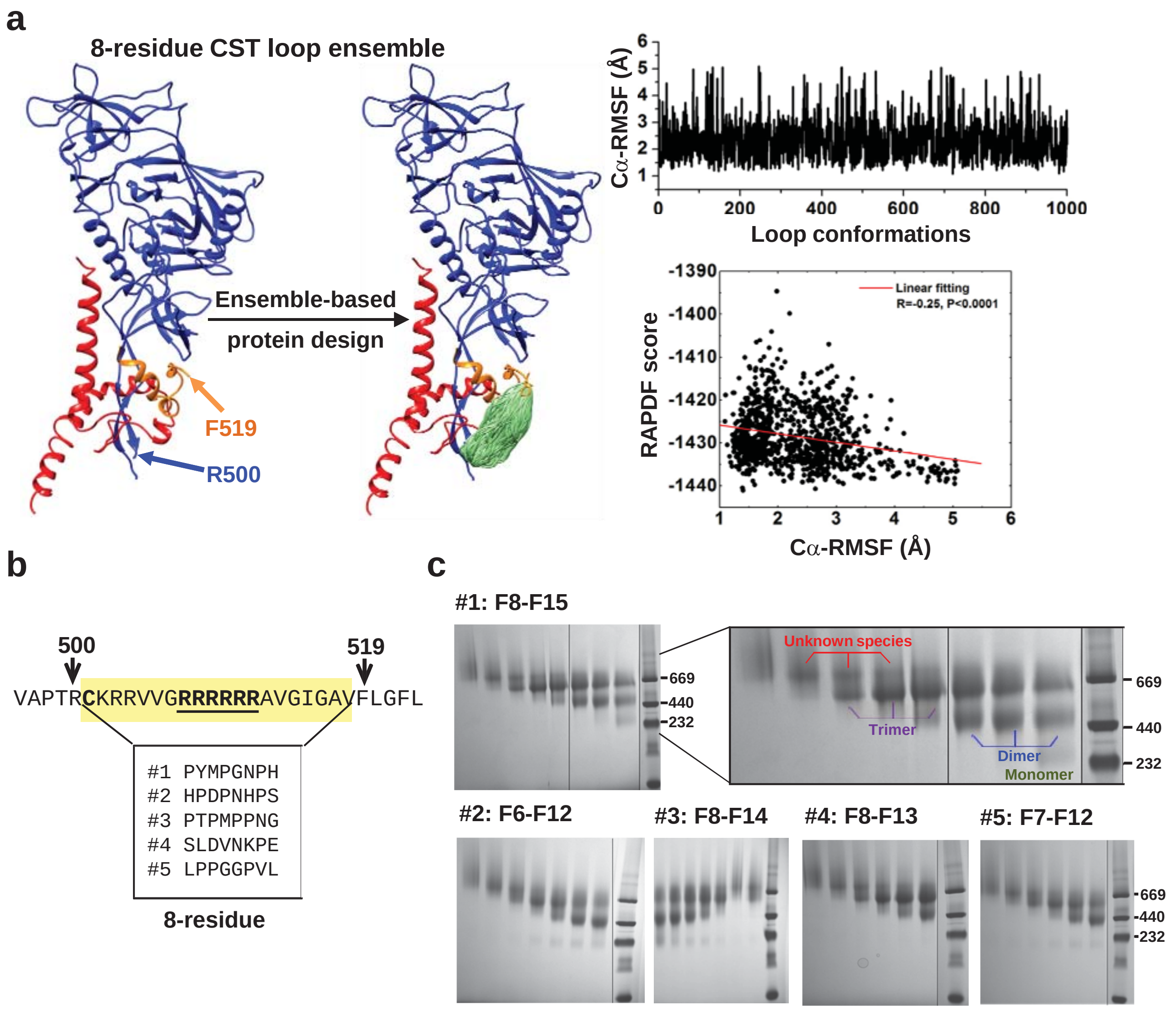

Supplementary Figure 7. Ensemble-based protein design of the cleavage site-containing region (500-519) and biochemical characterization of the top 5 designs. (**a**) Conformational ensemble of 8-residue loops connecting R500 and F519 (left), Cα RMSF distribution of 8-residue loops (upper right), and correlation between RAPDF score and Cα RMSF (lower right). (**b**) 5 top-ranking 8-residue cleavage site truncated (CST) redesigns. The region in WT BG505 SOSIP.664 that was subjected to computational design is highlighted in yellow. (**c**) BN-PAGE of five 293 F-expressed, GNL-purified BG505 CST proteins (CST1-5) after SEC on a Superdex 200 10/300 column. The SEC fractions tested are indicated above the gel images. For CST 1, the monomer, dimer and trimer bands, as well as an known Env species, are labeled on the BN gel.

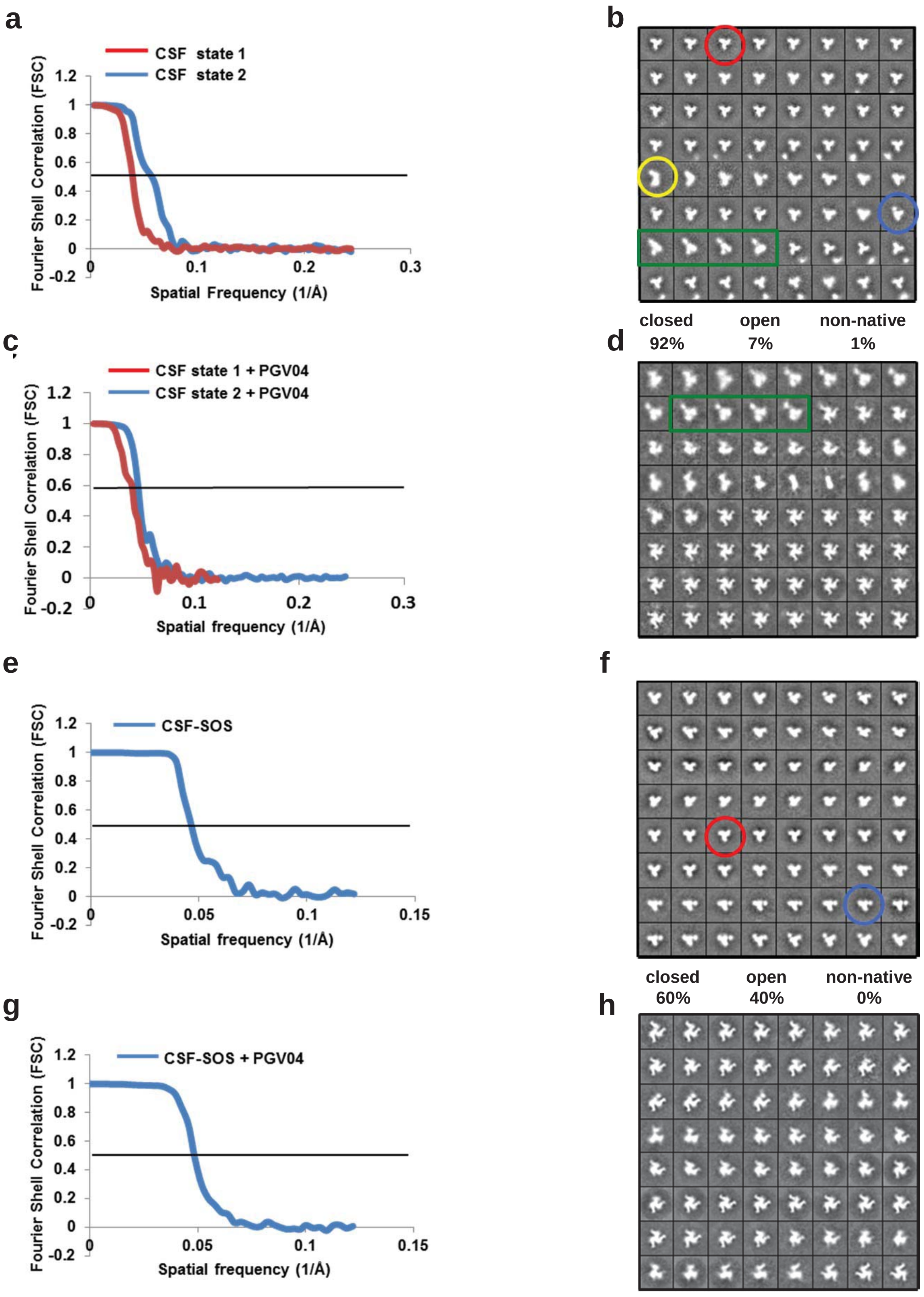
a
CSF state 1
CSF state 2
Fourier Shell Correlation (FSC)
Spatial Frequency (1/Å)
b
closed 92%
open 7%
non-native 1%
c
CSF state 1 + PGV04
CSF state 2 + PGV04
Fourier Shell Correlation (FSC)
Spatial frequency (1/Å)
d
e
CSF-SOS
Fourier Shell Correlation (FSC)
Spatial frequency (1/Å)
f
closed 60%
open 40%
non-native 0%
g
CSF-SOS + PGV04
Fourier Shell Correlation (FSC)
Spatial frequency (1/Å)
h

Supplementary Figure 8. Negative-stain EM of trimers based on HR1 redesign 1 and a near-full-length cleavage site linker (CSF). (**a**) The estimated resolution of the EM reconstructions were calculated from the Fourier shell correlation (FSC) using a cut-off of 0.5. The final estimated resolution for the CSF trimer was 17 Å in state 1 (red line) and 20 Å in state 2 (blue line). (**b**) Reference-free 2D class averages for the negative-stain EM of the CSF trimer. To analyze the quality of the trimers, we calculated the percentages of closed native-like trimers (red circle), partially open native-like trimers (blue circle), and non-native trimers (yellow circle). The side views of the trimer is indicated by the green square. (**c**) The final estimated resolution for the CSF trimer bound to PGV04 was 20 Å in state 1 (red line) and 21 Å in state 2 (blue line). (**d**) Reference-free 2D class averages for the negative-stain EM of the CSF trimer bound to PGV04. (**e**) The final estimated resolution for the CSF-SOS trimer was 21 Å. (**f**) Reference-free 2D class averages for the negative-stain EM of the CSF-SOS trimer. To analyze the quality of the trimers, we calculated the percentages of closed native-like trimers (red circle), partially open native-like trimers (blue circle), and non-native trimers. (**g**) The final estimated resolution for the CSF-SOS trimer bound to PGV04 was 20 Å. (**h**) Reference-free 2D class averages for the negative-stain EM of the CSF-SOS trimer bound to PGV04.

a

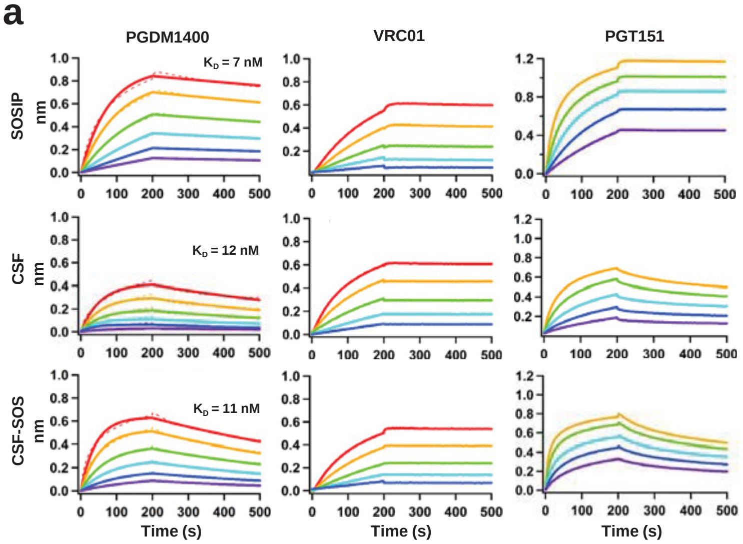

b

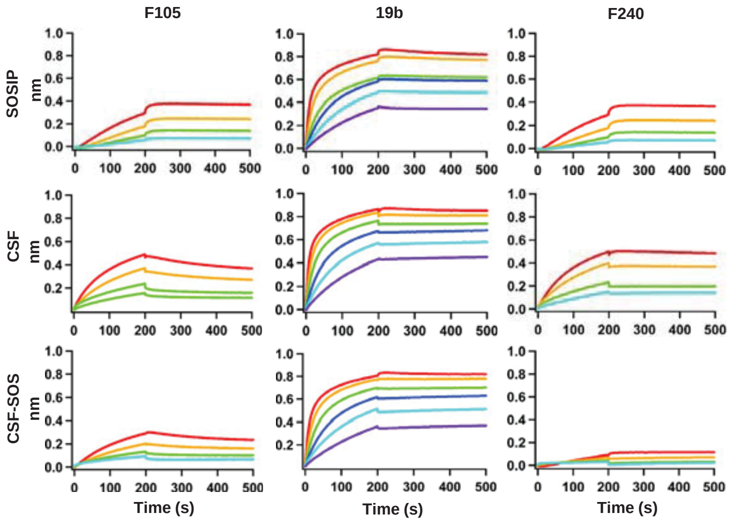

Supplementary Figure 9. Antigenic profiles for BG505 SOSIP.664 and two CSF trimers. Both CSF designs contain the same near-full-length linker at the cleavage site, with SOS indicating the disulfide bond (A501C/T605C) in CSF-SOS but not in CSF. Profiles of trimer binding to bNAbs and non-NAbs are shown in (**a**) and (**b**), respectively. Sensorgrams were obtained from an Octet RED96 instrument using a titration series of six trimer concentrations (200-12.5 nM by 2-fold dilution). K_D values calculated from 1:1 global fitting are labeled for the V1V2 apex-directed bNAb PGDM1400 in (a).

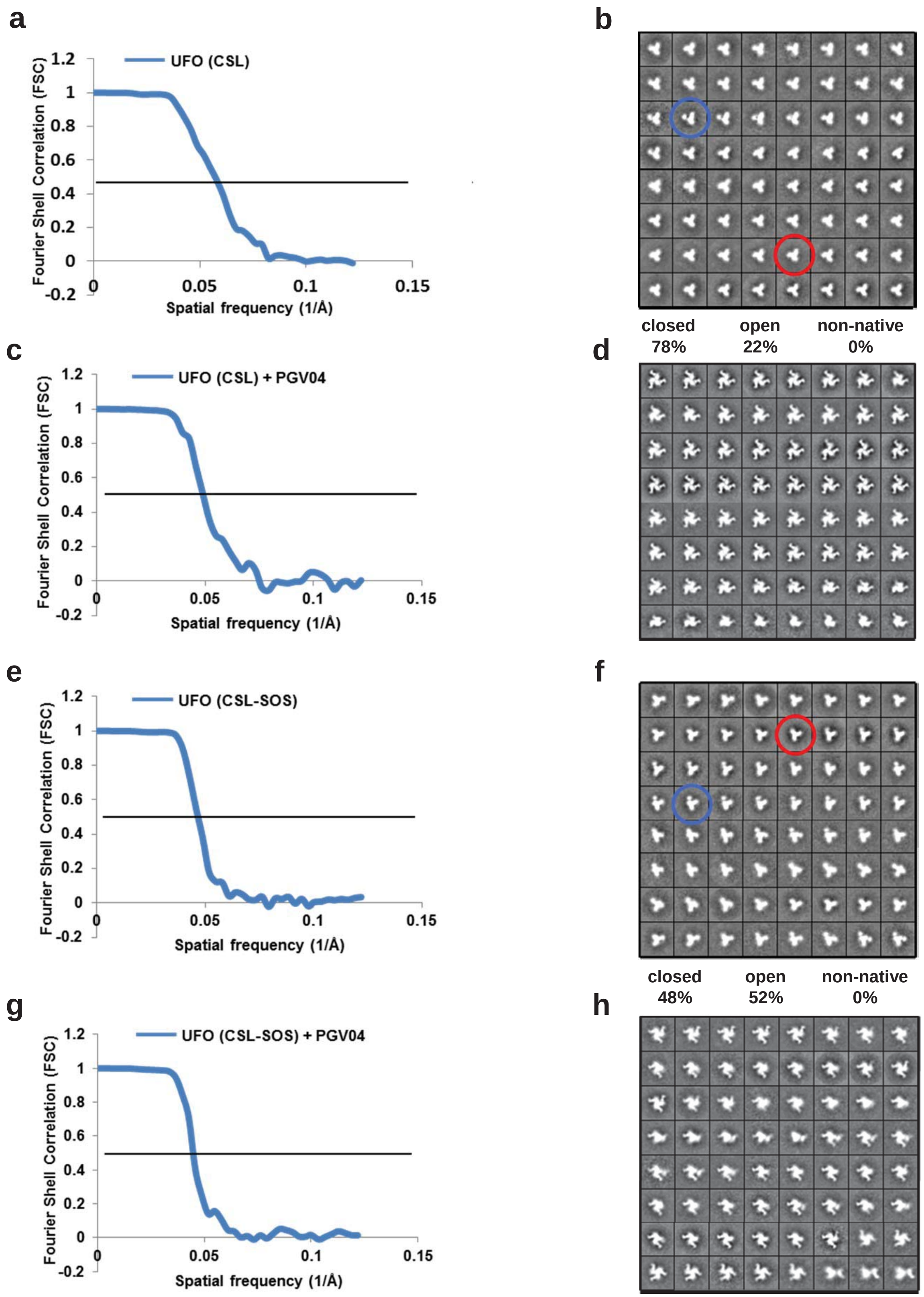
a
UFO (CSL)
Fourier Shell Correlation (FSC)
Spatial frequency (1/Å)
b
closed
78%
open
22%
non-native
0%
c
UFO (CSL) + PGV04
Fourier Shell Correlation (FSC)
Spatial frequency (1/Å)
d
e
UFO (CSL-SOS)
Fourier Shell Correlation (FSC)
Spatial frequency (1/Å)
f
closed
48%
open
52%
non-native
0%
g
UFO (CSL-SOS) + PGV04
Fourier Shell Correlation (FSC)
Spatial frequency (1/Å)
h

Supplementary Figure 10. Negative-stain EM of trimers based on HR1 redesign 1 and a long cleavage site linker (CSL). (**a**) The estimated resolutions of the EM reconstructions were calculated from the Fourier shell correlation (FSC) using a cut-off of 0.5. The final estimated resolution for the CSL trimer was 17 Å. (**b**) Reference-free 2D class averages of negative-stain EM of the CSL trimer. To analyze the quality of the trimers, we calculated the percentages of closed native-like trimers (red circle), partially open native-like trimers (blue circle), and non-native trimers. (**c**) The final estimated resolution for the CSL trimer bound to PGV04 was 20 Å. (**d**) Reference-free 2D class averages of negative-stain EM of the CSL trimer bound to PGV04. (**e**) The final estimated resolution for the CSL-SOS trimer was 21 Å. (**f**) Reference-free 2D class averages of negative-stain EM of the CSL-SOS trimer. To analyze the quality of the trimers, we calculated the percentages of closed native-like trimers (red circle), partially open native-like trimers (blue circle), and non-native trimers. (**g**) The final estimated resolution for the CSL-SOS trimer bound to PGV04 was 22 Å. (**h**) Reference-free 2D class averages of negative-stain EM of the CSL-SOS trimer bound to PGV04.

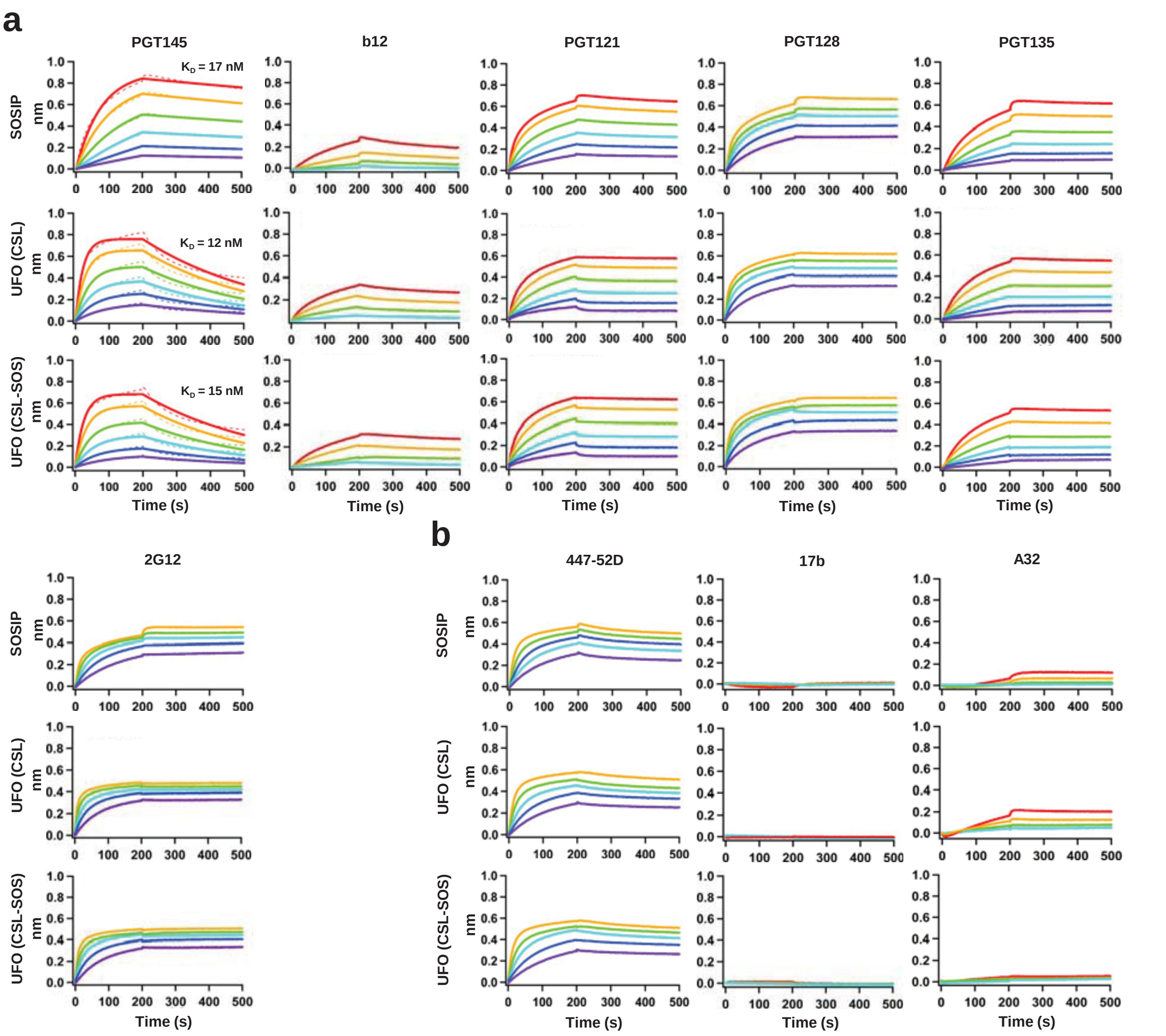

Supplementary Figure 11. Additional antigenic profiles for BG505 SOSIP.664 and two UFO CSL trimers. Both CSL designs contain the same NFL-like long linker at the cleavage site, with SOS indicating the disulfide bond (A501C/T605C) in CSL-SOS but not in CSL. Profiles of trimer binding to bNAbs and non-NAbs are shown in (**a**) and (**b**), respectively. Sensorgrams were obtained from an Octet RED96 instrument using a titration series of six trimer concentrations (200-12.5 nM by 2-fold dilution). K_D values calculated from 1:1 global fitting are labeled for the V1V2 apex-directed bNAb PGT145 in (a).

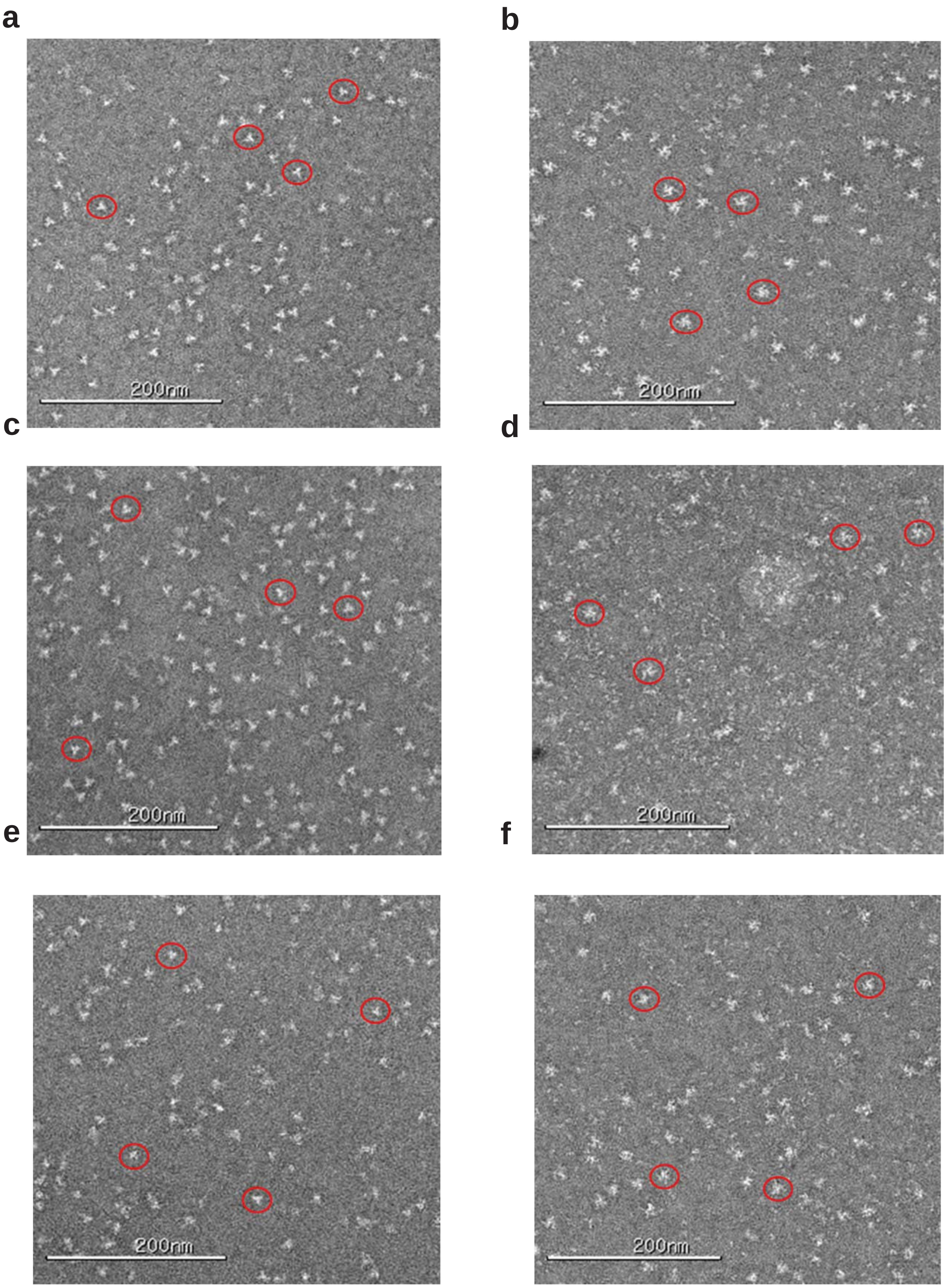
a
b
c
d
e
f
200nm
200nm
200nm
200nm
200nm
200nm

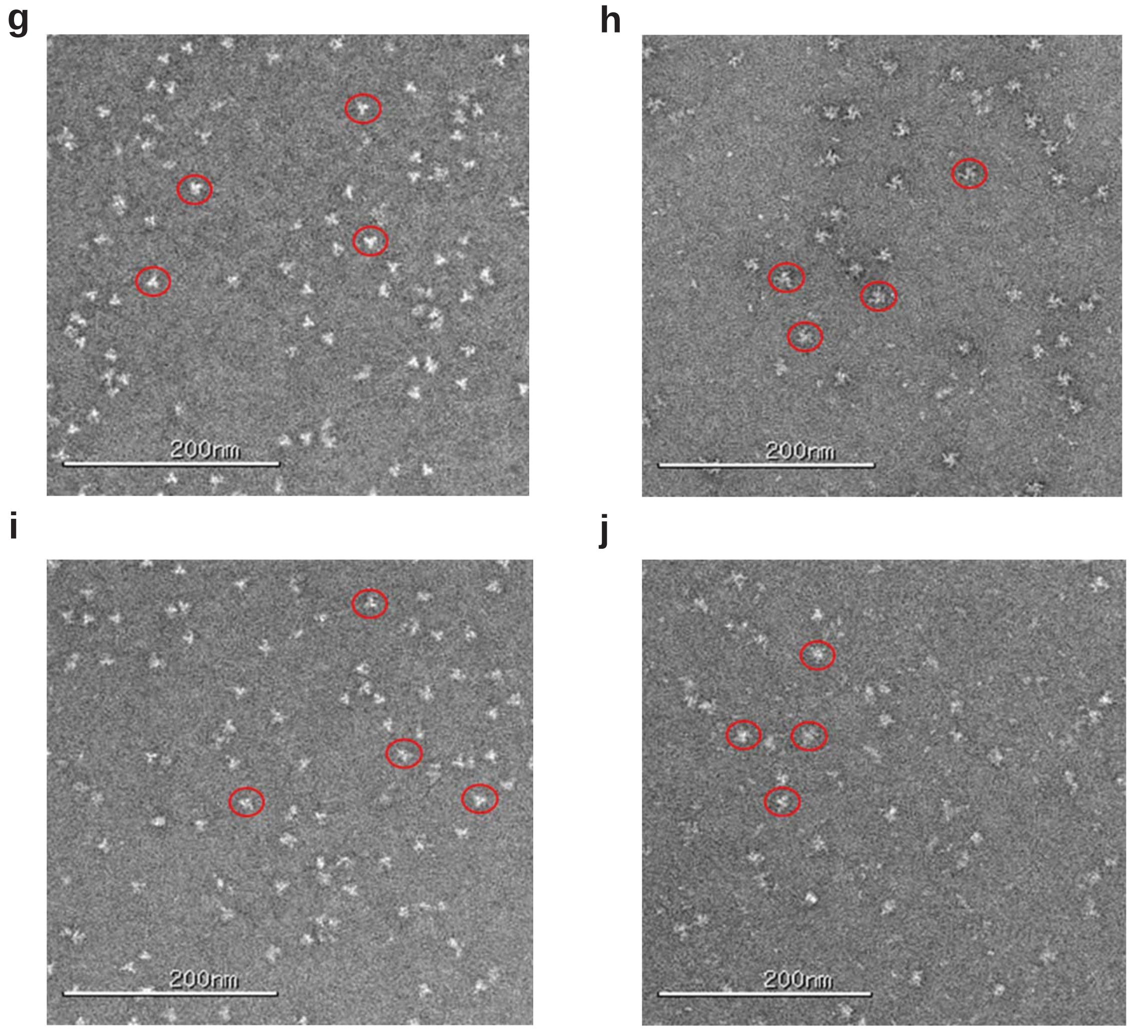

Supplementary Figure 12. Raw EM micrographs of HR1 redesign 1, CSF, and CSL trimers. (**a**) HR1 redesign 1 trimer unliganded, (**b**) HR1 redesign 1 trimer bound to PGV04, (**c**) CSF trimer unliganded, (**d**) CSF trimer bound to PGV04, (**e**) CSF-SOS trimer unliganded, (**f**) CSF-SOS trimer bound to PGV04, (**g**) CSL trimer unliganded, (**h**) CSL trimer bound to PGV04, (**i**) CSL-SOS trimer unliganded, and (**j**) CSL-SOS trimer bound to PGV04. Some individual particles are highlighted by red circles.

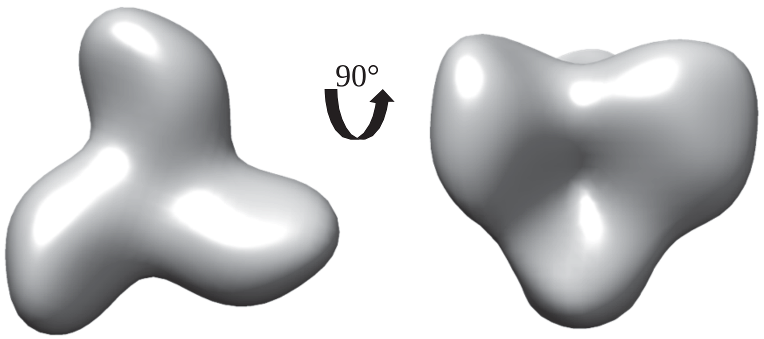

Supplementary Figure 13. Common lines model used for the refinement of all EM datasets. Top view (left) and side view (right) of WT BG505 SOSIP.664 gp140 trimer.

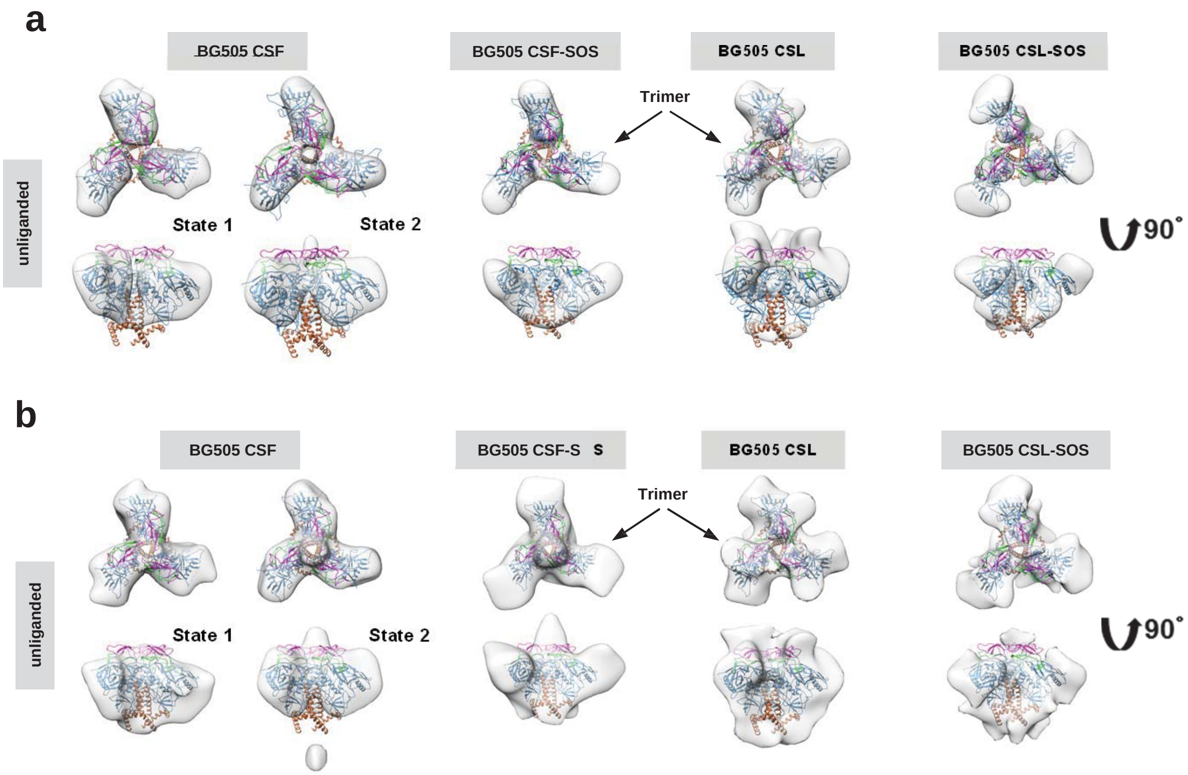

Supplementary Figure 14. EM densities of the CSF and CSL trimers derived from negative-stain EM. (**a**) EM densities of the unliganded trimers shown at a high contour level. CSF state1: 31, CSF state 2: 33, CSF-SOS: 68, CSL: 49, and CSL-SOS: 58. (**b**) EM densities of the unliganded trimers shown at a lower contour level, 20.

Supplementary Table 1. Number of particles collected and used for the different EM 3D reconstructions.

Trimer sample	Particles collected		Particles used for reconstruction	
HR1 redesign 1 unliganded	41956		18659	
HR1 redesign 1 bound to PGV04	35936		17959	
CSF unliganded	State 1	State 2	State 1	State 2
	8937	20854	5299	12365
CSF bound to PGV04	State 1	State 2	State 1	State 2
	11412	26628	3211	7494
CSF-SOS unliganded	52773		21684	
CSF-SOS bound to PGV04	30580		15206	
CSL unliganded	33451		13154	
CSL bound to PGV04	30019		12473	
CSL-SOS unliganded	30678		14848	
CSL-SOS bound to PGV04	32241		10544	
